# Unraveling the Link Between Serum Homocysteine Levels and Nutrient Deficiency in Subfertility: A Comprehensive Review

**DOI:** 10.7759/cureus.49296

**Published:** 2023-11-23

**Authors:** Swasti Shukla, Deepti Shrivastava

**Affiliations:** 1 Obstetrics and Gynecology, Acharya Vinoba Bhave Rural Hospital, Jawaharlal Nehru Medical College, Datta Meghe Institute of Higher Education and Research, Wardha, IND

**Keywords:** personalized medicine, serum levels, reproductive health, nutrient deficiencies, homocysteine, subfertility

## Abstract

Subfertility, a prevalent and emotionally taxing condition affecting couples worldwide, has garnered increasing attention in reproductive health. While its etiology remains multifaceted, emerging research has explored the role of serum homocysteine levels and nutrient deficiencies in influencing subfertility. This comprehensive review synthesizes current knowledge, beginning with an introduction to subfertility and the significance of investigating serum homocysteine levels. It proceeds to elucidate the role of nutrient deficiencies, particularly folate and vitamin B12, in homocysteine metabolism and examines existing research linking homocysteine to subfertility. The review explores potential mechanisms underlying this relationship, addressing the variability in study findings and their contributing factors. Implications for clinical practice, including assessing serum homocysteine levels, nutritional interventions, and personalized medicine, are discussed. Moreover, the review underscores the importance of ongoing research. It offers a call to action for advancing our understanding of subfertility and improving the lives of individuals and couples navigating reproduction challenges.

## Introduction and background

Infertility, defined as the inability to conceive after one year of regular, unprotected intercourse, is a distressing condition that affects a significant portion of the global population. In recent decades, subfertility, a related condition characterized by difficulty conceiving, has garnered increasing attention due to its widespread prevalence and profound impact on individuals and couples seeking to start a family. Subfertility is estimated to affect approximately 10-15% of couples worldwide, making it a prevalent reproductive health concern [1-3].

Subfertility encompasses a spectrum of fertility challenges, ranging from prolonged time to conception to recurrent pregnancy loss. Various factors, including hormonal imbalances, structural abnormalities, and lifestyle factors, can cause it. While advances in assisted reproductive technologies have provided hope and options for many subfertile couples, much remains unknown about the underlying causes of subfertility. This knowledge gap underscores the need for comprehensive research into the factors contributing to subfertility [3-5]. In recent years, emerging evidence has suggested a potential link between elevated serum homocysteine levels and subfertility. Homocysteine, a sulfur-containing amino acid, plays a crucial role in various biochemical pathways within the body. Elevated homocysteine levels have been widely recognized as a risk factor for cardiovascular disease, neurodegenerative disorders, and adverse pregnancy outcomes, among other health concerns. However, its connection to subfertility remains a relatively unexplored area of research [6,7].

Understanding the role of serum homocysteine levels in subfertility is significant for several reasons. Firstly, it offers a novel perspective on the intricate interplay between metabolic factors and reproductive health. Secondly, if a link is established, it may provide a non-invasive and cost-effective biomarker for assessing subfertility risk. Lastly, elucidating the relationship between homocysteine and subfertility could open avenues for targeted interventions, potentially improving the chances of conception for affected individuals and couples [8]. This comprehensive review aims to unravel the complex relationship between serum homocysteine levels and nutrient deficiency in the context of subfertility. We aim to critically analyze existing clinical and experimental literature to assess the extent to which serum homocysteine levels may be associated with subfertility. Furthermore, we will explore the potential underlying mechanisms through which nutrient deficiencies, particularly vitamins such as folate and B12, may influence homocysteine levels and, consequently, reproductive outcomes.

## Review

Nutrient deficiency and subfertility

Definition of Subfertility

Subfertility, often used interchangeably with "unexplained infertility," refers to a condition in which couples experience difficulty conceiving despite engaging in regular, unprotected sexual intercourse for an extended period, typically one year or more. Unlike infertility, where a clear cause for the inability to conceive can be identified, subfertility encompasses cases where no specific, evident reason for the delay in conception can be pinpointed [9].

Common Causes and Risk Factors

Hormonal imbalances: Hormones play a big role in the menstrual cycle and reproduction. Problems with hormones like estrogen, progesterone, and luteinizing hormone (LH) can lead to irregular periods, lack of ovulation, and fertility issues. Conditions like polycystic ovary syndrome (PCOS) involve hormonal imbalances that can make it harder to get pregnant [10].

Structural abnormalities: Issues with the reproductive organs can block successful fertilization. Conditions like uterine fibroids, polyps, or blocked fallopian tubes can stop sperm from reaching the egg or prevent embryo implantation. Identifying and fixing these structural problems to improve fertility [9].

Lifestyle factors: Choices in lifestyle can strongly affect fertility. Smoking, too much alcohol, and substance abuse can reduce fertility in both men and women. Obesity can mess with hormones and ovulation, posing another risk. Also, a poor diet and insufficient nutrients can harm reproductive health [11].

Age: A woman's age is crucial for fertility. Fertility decreases as a woman gets older, mainly because of fewer and lower-quality eggs. After 35, fertility declines more rapidly. Being older during pregnancy increases the chances of fertility problems and pregnancy complications [12].

Psychological factors: Mental factors like stress, anxiety, and depression can seriously impact fertility. Chronic stress, for example, can mess up the delicate hormonal balance needed for ovulation and successful pregnancy. Also, psychological issues can affect sexual function and desire, changing how often couples have sex and influencing fertility [13].

The Role of Nutrition in Reproductive Health

Nutrition is pivotal in reproductive health, influencing various aspects crucial for fertility and overall well-being. Folate (vitamin B9) is a critical nutrient known for its significance before and during pregnancy. Essential for DNA synthesis and gene regulation through methylation processes, adequate folate intake is vital to prevent neural tube defects in the developing fetus. Women of childbearing age are advised to ensure sufficient folate intake through diet or supplementation to support a healthy pregnancy [14-16].

Vitamin B12, another essential B vitamin, is crucial for DNA synthesis and maintaining healthy nerve cells. Deficiency in vitamin B12 can lead to anemia, reduced red blood cell production, and neurological issues. Proper vitamin B12 status is important for overall health and may impact fertility [15-16]. Vitamin D affects hormonal balance, immune system regulation, and calcium absorption, playing a role in maintaining the health of various body systems, including the reproductive system. Insufficient vitamin D levels may be associated with subfertility, emphasizing the importance of ensuring adequate intake and exposure to sunlight for reproductive health [17-20].

Iron, crucial for forming red blood cells and oxygen transport, maintains optimal hemoglobin levels. Iron deficiency can lead to anemia, disrupting menstrual cycles and affecting fertility by disturbing the delicate balance of hormones. Adequate iron intake is crucial for sustaining reproductive health, especially in women with heavy menstrual bleeding [17]. Omega-3 fatty acids, particularly DHA and EPA, are known for their anti-inflammatory properties, potentially influencing reproductive health by regulating menstrual cycles and reducing inflammation [18-21].

Serum homocysteine levels: an emerging biomarker

What Is Homocysteine?

Homocysteine is a sulfur-containing amino acid that is an intermediate in methionine metabolism, another amino acid obtained from dietary protein sources. It is produced during the demethylation of methionine, and various factors, including dietary intake of certain nutrients, genetic variations, and metabolic processes, influence its levels in the body. Homocysteine exists in two forms: free homocysteine (not bound to proteins) and protein-bound homocysteine [22].

Biological Functions and Metabolism of Homocysteine

Methylation reactions are fundamental processes where homocysteine serves as a key participant, acting as a provider of methyl groups (CH3) during the transfer to various target molecules. This transfer of methyl groups is not a mere biochemical detail; it is a highly significant mechanism with far-reaching consequences for cellular functioning. Through methylation, homocysteine actively contributes to modifying essential molecules within the cell. These modifications are not arbitrary but are intricately tied to the regulation and optimization of cellular processes. The outcomes of methylation reactions extend beyond a singular event; they create a cascade of molecular changes that influence cellular functions critical to overall health. In essence, the role of homocysteine in methylation reactions is a dynamic and vital aspect of cellular biochemistry. It underscores the interconnected nature of biochemical processes and emphasizes a single molecule's profound impact on the intricate dance of molecular modifications within the cell. Understanding these methylation reactions provides insight into the broader landscape of cellular health and functioning [23].

Gene regulation: Methylation occupies a central position in the orchestration of gene expression. DNA methylation entails adding methyl groups to specific regions of DNA, effectively governing whether particular genes are activated or silenced. This regulatory mechanism controls various cellular functions, encompassing cell differentiation, growth, and responsiveness to environmental cues. The involvement of homocysteine in methylation reactions endows it with an indirect role in the modulation of gene expression, thereby holding the potential to impact health and disease outcomes [24].

Neurotransmitter and hormone metabolism: Methylation proves indispensable in the metabolism of neurotransmitters (e.g., dopamine, serotonin) and hormones (e.g., epinephrine, norepinephrine). These molecules assume critical roles in mood regulation, cognitive processes, and the body's response to stress. Homocysteine's active participation in methylation reactions assumes significance in ensuring the proper synthesis and metabolism of these bioactive compounds, thereby influencing mood, cognition, and stress response mechanisms [25].

Transsulfuration pathway: Homocysteine is pivotal in the transsulfuration pathway, another vital metabolic route within the body. This pathway facilitates the conversion of homocysteine into cysteine, an amino acid with multifaceted functions. Cysteine is a precursor for synthesizing glutathione, one of the body's most potent antioxidants [26].

Antioxidant defense: Glutathione emerges as a linchpin in shielding cells from the detrimental effects of oxidative stress. Oxidative stress occurs when there is an imbalance between reactive oxygen species (ROS) and the body's antioxidant defenses, leading to cellular damage. Glutathione, a critical antioxidant, counteracts the harmful effects of ROS and maintains the delicate balance of redox processes within cells. Homocysteine's involvement in cysteine synthesis indirectly bolsters glutathione production, fortifying the body's capacity to fend off oxidative stress and uphold cellular health [27].

Role of Homocysteine in Cardiovascular Health

Elevated homocysteine levels have been associated with impaired endothelial function, and the mechanism through which this occurs is noteworthy. The endothelium, which forms the inner lining of blood vessels, plays a crucial role in regulating blood flow and maintaining vascular health. When homocysteine levels are elevated, they can adversely affect the endothelium. The intricate mechanism involves disrupting the function of the endothelium, leading to diminished vasodilation, the widening of blood vessels, and an increase in inflammation. This disruption, caused by elevated homocysteine, contributes to endothelial dysfunction. As a result, there is a compromise in the normal functioning of blood vessels, potentially leading to high blood pressure and an elevated risk of cardiovascular events [28].

Oxidative stress: Homocysteine has been shown to promote oxidative stress within the vascular system. Oxidative stress occurs when there is an imbalance between free radicals (reactive molecules) and antioxidants in the body. Elevated homocysteine levels can contribute to oxidative stress, which, in turn, can damage blood vessel walls. This damage can trigger an inflammatory response and promote the development of atherosclerosis, a condition characterized by the buildup of plaques in the arteries. Atherosclerosis is a major risk factor for heart disease and stroke [29].

Thrombosis: High levels of homocysteine may enhance the formation of blood clots, a process known as thrombosis. However, the specific threshold at which homocysteine levels begin to disrupt the normal regulation of blood clotting mechanisms and potentially lead to an increased tendency to form blood clots is not well-established. This heightened risk of thrombosis can contribute to cardiovascular events, such as heart attacks and strokes [30].

Nutrients and homocysteine metabolism

Folate (Vitamin B9) and Its Role in Homocysteine Metabolism

Methionine synthesis: Folate is required as a co-factor for the enzyme methionine synthase, which is central to the conversion of homocysteine into methionine. This enzymatic reaction is critical for maintaining optimal homocysteine levels in the body. Methionine, the end product of this reaction, is an essential amino acid necessary for protein synthesis, including the production of enzymes and structural proteins. Methionine is also a precursor for S-adenosylmethionine (SAMe), a molecule involved in various biochemical reactions, including DNA methylation [31].

Protein and DNA synthesis: Methionine generated through the methionine synthase pathway is indispensable for synthesizing proteins and DNA. In the context of reproductive health, the availability of methionine and its subsequent use in protein and DNA synthesis are essential for various processes, including the development and maturation of ova, the production of sperm, and the proper functioning of reproductive organs [32].

DNA methylation: Folate also plays a crucial role in DNA methylation, which involves adding methyl groups to DNA molecules. DNA methylation is a fundamental mechanism of gene regulation that can influence the expression of genes associated with various aspects of health, including reproduction. Proper DNA methylation patterns are essential for the precise control of gene expression during critical reproductive processes, such as gametogenesis (sperm and egg formation), fertilization, and embryonic development [33].

Gene regulation in reproduction: DNA methylation patterns can impact the expression of genes involved in reproductive health, such as those related to hormone regulation, gamete quality, and embryonic development. Disruptions in DNA methylation patterns due to folate deficiency may lead to suboptimal gene regulation in these critical reproductive processes [34].

Vitamin B12 and Its Impact on Homocysteine Levels

Vitamin B12 is a vital co-factor for the enzyme methionine synthase and folate, playing a central role in homocysteine metabolism. This enzymatic process is essential for converting homocysteine into methionine, a crucial amino acid for various biological functions [35]. Both folate and vitamin B12 are necessary to effectively function methionine synthase. Beyond its involvement in homocysteine metabolism, vitamin B12 holds critical importance in maintaining the proper functioning of the nervous system. Vitamin B12 deficiency can manifest in neurological symptoms, including peripheral neuropathy (damage to peripheral nerves) and cognitive impairments. Given the integral role of the nervous system in regulating reproductive processes, such as hormonal regulation, ovulation, and sperm production, disruptions in neurological function may have a notable impact on fertility [36].

Other Vitamins and Minerals Affecting Homocysteine

Vitamin B6 (pyridoxine): Vitamin B6 is a vital player in homocysteine metabolism. It functions as a co-factor for enzymes involved in homocysteine metabolism, particularly in converting homocysteine to cystathionine. Through this enzymatic activity, vitamin B6 assists in the transformation of homocysteine into a less harmful compound, cystathionine. Adequate vitamin B6 levels are necessary to facilitate this enzymatic reaction effectively. A deficiency in vitamin B6 can disrupt homocysteine metabolism, potentially leading to elevated homocysteine levels, which may have implications for subfertility [37].

Riboflavin (vitamin B2): While not directly involved in homocysteine metabolism, riboflavin plays an important indirect role. Riboflavin is essential for activating folate and vitamin B6, two key players in the homocysteine metabolic pathway. Riboflavin is a co-factor for enzymes that convert folate and vitamin B6 into active forms required for homocysteine metabolism. Thus, riboflavin deficiency can indirectly impact homocysteine metabolism by limiting the activation of folate and vitamin B6, potentially leading to elevated homocysteine levels [38].

Betaine: Betaine is a naturally occurring compound found in certain foods, including beets, spinach, whole wheat, and seafood. It has a unique role in homocysteine metabolism, distinct from the traditional pathways involving folate and vitamin B12. Betaine can participate in the remethylation of homocysteine to methionine independently of these vitamins. This alternative pathway for homocysteine metabolism is particularly important in cases where deficiencies in folate or vitamin B12 are present or genetic variations affect traditional homocysteine metabolism pathways. The availability of betaine from dietary sources may influence the efficiency of this alternative pathway and, subsequently, homocysteine levels [39].

Dietary Sources of These Nutrients

Meeting the nutritional requirements for folate, vitamin B12, B6, riboflavin (B2), and betaine is essential for maintaining homocysteine metabolism and overall reproductive health, as described in Table 1 [40].

**Table 1:** Dietary sources of nutrients The author self-created the table.

Nutrient	Dietary Sources
Folate (vitamin B9)	Leafy green vegetables (e.g., spinach, kale), legumes (e.g., lentils, chickpeas), citrus fruits (e.g., oranges), fortified cereals
Vitamin B12	Meat (especially liver and fish), dairy products, eggs
Vitamin B6	Poultry (chicken and turkey), fish (salmon, tuna, trout), bananas, potatoes (especially with the skin), fortified cereals
Riboflavin (vitamin B2)	Dairy products (milk, yogurt, cheese), lean meats (beef, pork, chicken), green leafy vegetables (spinach, broccoli, asparagus), whole grains (whole wheat bread, brown rice, fortified cereals)
Betaine	Beets (both the root and leaves), spinach, whole wheat (e.g., whole wheat bread, pasta), seafood (e.g., shrimp, cod)

Serum homocysteine levels in subfertility

An expanding body of research has investigated the potential association between serum homocysteine levels and subfertility. These studies have explored various facets of this relationship, shedding light on the prevalence of elevated homocysteine in subfertility patients, potential mechanisms that link homocysteine to subfertility, and the impact of nutrient deficiencies on homocysteine levels. A comprehensive review of these studies offers valuable insights into the role of homocysteine in the context of subfertility [41].

Prevalence of Elevated Homocysteine

The incidence of elevated homocysteine levels exhibits variability based on the specific population under examination and the criteria employed to define elevated homocysteine. In a cross-sectional study conducted in Hunan, China, the prevalence of hyperhomocysteinemia was notably high, reaching 35.4% [41]. The general populace's estimated occurrence of mild hyperhomocysteinemia falls within 5% to 7% [41]. Among hypertensive individuals aged 35 and older, the prevalence of hyperhomocysteinemia was determined to be 36.1% [41]. Utilizing administrative claims data, a separate study gauged the prevalence of homocystinuria in the United States, revealing an approximate incidence of 1 in 10,000 individuals [41]. Conversely, another investigation projected the prevalence of diagnosed homocystinuria in the US to span between 12,113 and 31,162 cases [5]. In an observational cross-sectional study focusing on an elderly demographic, while the prevalence of total hyperhomocysteinemia was not explicitly disclosed, the findings underscored a significant age-related increase in homocysteine levels [41]. These diverse prevalence figures highlight the importance of considering both demographic factors and the specific criteria employed when interpreting the incidence of elevated homocysteine across different populations.

Potential Mechanisms

Research has delved into mechanisms that might explain the relationship between homocysteine and subfertility. These mechanisms include endothelial dysfunction, oxidative stress, hormonal disruption, inflammation, and alterations in DNA methylation patterns. Understanding these mechanisms at the cellular and molecular levels is critical for comprehending how elevated homocysteine might affect reproductive health [41].

Nutrient Deficiencies

Studies have investigated the impact of nutrient deficiencies, particularly folate, vitamin B12, and vitamin B6, on serum homocysteine levels in subfertility patients. Nutrient deficiencies can disrupt homocysteine metabolism, potentially contributing to elevated levels. Exploring the prevalence of these deficiencies and their role in subfertility helps establish a more comprehensive understanding of the factors influencing homocysteine in this context [41].

Outcome Measures

Researchers have assessed various outcome measures in their studies, including time to conception, live birth rates, pregnancy complications, and neonatal outcomes, to determine the clinical significance of elevated homocysteine levels in subfertility patients. These outcome measures provide insights into the potential impact of homocysteine on fertility and reproductive success [42].

Correlation Between Serum Homocysteine Levels and Subfertility

High homocysteine levels have been associated with infertility and pregnancy complications such as preeclampsia, preterm delivery, miscarriage, and low birth weight [43]. Several studies have investigated the association between homocysteine levels and infertility. High homocysteine levels have been linked to poor oocyte quality, male infertility due to abnormal sperm, and unexplained infertility in vitro fertilization/intracytoplasmic sperm injection (IVF/ICSI) [44-45]. One study found that lower homocysteine levels in embryo culture medium were associated with a better chance of pregnancy and better embryo quality [45]. However, another study found no association between serum homocysteine levels and IVF/ICSI pregnancy outcomes [46]. Additionally, high homocysteine levels have been identified as a risk factor for unexplained infertility and pregnancy complications such as preeclampsia, preterm delivery, miscarriage, and low birth weight [3]. Aging and high levels of plasma homocysteine are also strongly associated with thrombotic events during IVF [43].

Potential Mechanisms Linking Homocysteine to Subfertility

Several studies have explored the link between homocysteine levels and subfertility. Elevated homocysteine levels have been associated with various aspects of infertility, including poor oocyte quality, male infertility due to abnormal sperm, and unexplained infertility in vitro fertilization/intracytoplasmic sperm injection (IVF/ICSI) [43-44]. Lower follicular fluid homocysteine levels have been associated with a better chance of clinical pregnancy, and homocysteine-lowering agents have shown a favorable impact on the outcome of infertility in cases of unexplained infertility associated with hyperhomocysteinemia [45-46]. Additionally, high homocysteine levels have been identified as a risk factor for pregnancy complications such as preeclampsia, preterm delivery, miscarriage, and low birth weight [46]. These findings suggest a potential role for homocysteine in assessing and managing subfertility.

Endothelial dysfunction: Elevated homocysteine levels impair endothelial function, leading to endothelial dysfunction. This dysfunction can reduce blood flow to various organs, including the reproductive organs. Reduced blood flow to these organs may affect their function and compromise fertility. Proper blood flow is essential for nourishing and oxygenating reproductive tissues, making endothelial dysfunction a potential contributor to subfertility [47-48].

Oxidative stress: Homocysteine has been linked to promoting oxidative stress in the body. Oxidative stress occurs when an imbalance between free radicals and antioxidants leads to cellular damage. Elevated homocysteine levels can contribute to oxidative stress, adversely affecting ova and sperm quality. Increased oxidative stress can damage these reproductive cells' DNA, reducing their viability and fertility potential [49].

Hormonal disruption: Elevated homocysteine levels may interfere with hormonal balance, including regulating sex hormones. Proper hormonal balance is essential for various aspects of fertility, including the development and release of eggs, the quality of sperm, and the receptivity of the uterine lining for embryo implantation. Therefore, disruptions in hormonal signaling pathways could affect fertility [50].

Inflammation: Elevated homocysteine levels have been associated with increased inflammation in the body. Chronic inflammation can have systemic effects and disrupt normal reproductive processes. Inflammation may impair the function of the reproductive organs, alter the balance of immune cells in the reproductive tract, and negatively impact the quality of eggs and sperm [51].

DNA methylation: Homocysteine can influence epigenetic processes, particularly DNA methylation patterns. Alterations in DNA methylation can affect gene expression, including genes critical for reproductive health. Changes in the epigenetic landscape may lead to suboptimal regulation of genes involved in fertility and reproductive processes [52].

Variability in Study Findings and Factors Contributing to It

Study populations: Variations in study populations can significantly impact the strength of the association between serum homocysteine levels and subfertility. Age, sex, and underlying health conditions can differ between study cohorts. For example, homocysteine and subfertility may be more pronounced in older individuals or those with specific medical conditions that affect homocysteine metabolism [53].

Nutritional status: Differences in the nutritional status of study participants, particularly in terms of folate, vitamin B12, and vitamin B6 intake, can affect homocysteine levels and complicate the interpretation of results. Nutrient deficiencies can vary across populations and influence homocysteine levels differently. Studies should account for these variations by assessing and reporting participants' nutritional status [54].

Methodological differences: Variations in study design and methodology can contribute to discrepancies in findings. Differences in sample size, follow-up duration, and measurement methods for homocysteine levels may impact the ability to detect associations accurately. Standardization of study protocols and careful consideration of statistical methods are essential to minimize methodological biases [55].

Genetic factors: Genetic variations in enzymes involved in homocysteine metabolism can impact individual homocysteine levels and responses to nutrient deficiencies. Polymorphisms in genes encoding enzymes such as MTHFR can influence homocysteine metabolism and may interact with nutritional factors. Studies should consider genetic factors as potential effect modifiers and stratify analyses accordingly [56].

Other confounding factors: Lifestyle factors can influence homocysteine levels and contribute to variability in study results. Smoking, excessive alcohol consumption, and BMI are examples of factors that can affect homocysteine metabolism. It is essential to account for these confounding variables in study design and analysis to isolate the specific impact of homocysteine on subfertility [57].

Implications for clinical practice

Assessing Serum Homocysteine Levels in Subfertility Patients

Diagnostic value: Routine measurement of serum homocysteine levels can be a valuable diagnostic tool in subfertility. Elevated homocysteine levels may signal underlying nutrient deficiencies or metabolic imbalances that could contribute to subfertility. While elevated homocysteine alone may not be the sole cause of subfertility, it can indicate potential underlying issues that warrant further investigation and intervention [58].

Patient selection: Healthcare providers should consider including serum homocysteine assessment as part of the diagnostic workup for subfertility, particularly in cases with unexplained subfertility or other risk factors for elevated homocysteine. Risk factors may include a history of nutrient deficiencies, certain medical conditions (e.g., malabsorption disorders), or lifestyle factors (e.g., smoking, excessive alcohol consumption) that can affect homocysteine metabolism. Identifying individuals at risk for elevated homocysteine levels can help tailor diagnostic and treatment approaches [42].

Interpretation: Interpreting homocysteine levels should be done with other clinical information. A comprehensive evaluation should include a thorough medical history to identify potential risk factors and assess nutrient status, particularly folate and vitamin B12 levels, integral to homocysteine metabolism. Evaluating other potential causes of subfertility, such as hormonal imbalances and anatomical factors, is essential to establish a comprehensive diagnosis and treatment plan [47].

Reference ranges: Establishing appropriate reference ranges for serum homocysteine levels in the context of subfertility is essential. Optimal homocysteine levels may differ from general population reference values due to the unique considerations in subfertility patients. Healthcare providers should work collaboratively with laboratories to determine relevant reference ranges that account for factors specific to subfertility, such as nutritional status and underlying medical conditions [59].

Nutritional Interventions to Regulate Homocysteine Levels

Supplementation: Clinicians may consider recommending supplementation for individuals with confirmed nutrient deficiencies, specifically in folate or vitamin B12. These nutrients play essential roles as cofactors in homocysteine metabolism. Supplementation is advisable to address deficiencies and facilitate the conversion of homocysteine to methionine. Dosage and duration should be personalized based on ongoing monitoring of nutrient status and homocysteine levels [60].

Dietary modification: Clinicians must advise subfertility patients to adopt a balanced diet rich in folate, vitamin B12, and other essential nutrients to optimize homocysteine metabolism. Dietary sources such as leafy green vegetables, legumes, meat, fish, dairy, and fortified foods can help maintain adequate nutrient levels and support homocysteine metabolism. Personalized dietary recommendations, based on individual nutritional status and preferences, can be provided by nutritionists [61].

Lifestyle changes: Recognizing the association between lifestyle factors and elevated homocysteine levels, healthcare providers should actively address and modify habits like smoking and excessive alcohol consumption in subfertility patients. Integrating smoking cessation programs and alcohol reduction strategies into the treatment plan can help mitigate the impact of these modifiable risk factors on homocysteine levels. These lifestyle modifications benefit homocysteine metabolism and promote overall reproductive health [62].

Monitoring: Clinicians should emphasize the importance of regularly monitoring homocysteine levels during nutritional interventions. This enables healthcare providers to assess treatment efficacy and make necessary adjustments. Individualized monitoring, based on the patient's initial homocysteine levels and response to interventions, ensures effective resolution of nutrient deficiencies and maintenance of homocysteine levels within the target range [63].

Case Studies and Clinical Trials Demonstrating Efficacy

Case studies: Documenting individual case studies where nutritional interventions successfully regulated homocysteine levels and led to improved fertility outcomes can provide valuable real-world insights. These cases should highlight the specific nutritional deficiencies identified, the interventions employed (e.g., dietary changes, supplementation), and the resulting changes in homocysteine levels and fertility outcomes. Case studies offer anecdotal evidence that can inspire similar approaches in clinical practice and serve as preliminary evidence of effectiveness [8].

Clinical trials: Rigorous clinical trials are essential to systematically assess the effectiveness of specific nutritional interventions on subfertility patients with elevated homocysteine levels. To ensure scientific rigor, well-designed trials should include appropriate control groups (e.g., placebo or standard treatment) and randomization. Furthermore, these trials should incorporate long-term follow-up to evaluate reproductive success as a primary outcome measure. Key parameters include time to conception, live birth rates, pregnancy complications, and neonatal outcomes. Trials should also stratify patients based on the degree of homocysteine elevation to explore potential dose-response relationships [64].

Recommendations for Healthcare Providers

Comprehensive assessment: Conduct a comprehensive evaluation of subfertility patients. This should encompass a thorough medical history, physical examination, and assessment of nutritional status. A detailed medical history helps identify potential risk factors and underlying conditions contributing to subfertility. A physical examination can reveal physical factors affecting fertility, and assessing nutritional status, including serum homocysteine levels, is crucial to identify nutrient deficiencies that may be amenable to intervention [65].

Individualized approach: Recognize that subfertility is a complex condition with diverse underlying causes. Adopt an individualized approach to diagnosis and treatment, considering each patient's specific needs and circumstances. Age, medical history, lifestyle, and partner factors should all inform the diagnostic and treatment plan. Individualization ensures that interventions are tailored to address the unique factors contributing to subfertility, potentially improving treatment success [66].

Multidisciplinary collaboration: Subfertility often involves multifaceted factors, including nutritional, hormonal, and reproductive aspects. Collaborate with a multidisciplinary team of healthcare professionals, including nutritionists, reproductive endocrinologists, genetic counselors, and other specialists. This collaborative approach allows for a comprehensive assessment of subfertility patients, facilitates the development of holistic treatment plans, and ensures patients receive the most appropriate and effective interventions [67].

Patient education: Educate subfertility patients about the importance of nutrition in reproductive health. Patients should be informed about the potential role of serum homocysteine levels and nutrient deficiencies in subfertility. Guide on maintaining a balanced diet, addressing nutrient deficiencies, and making lifestyle changes that support reproductive health. Informed patients are more likely to actively participate in their treatment and make positive changes to enhance their fertility [68].

Long-term care: Recognize that subfertility management often requires ongoing care and monitoring. Implement a long-term care plan for subfertility patients to assess treatment progress and adjust interventions as necessary. Regular monitoring of serum homocysteine levels, nutritional status, and reproductive outcomes allows for the timely identification of treatment effectiveness and the need for modifications. Continuity of care is essential to support patients throughout their subfertility journey [69].

Future directions and research gaps

Areas Requiring Further Investigation

Causality: Whether elevated homocysteine levels directly contribute to subfertility or are merely a marker of underlying metabolic disturbances remains a pivotal question. Establishing causality is complex due to the multifactorial nature of subfertility. Future research should employ longitudinal and interventional study designs to decipher whether elevated homocysteine plays a causal role in subfertility or is an indirect consequence of underlying conditions such as nutrient deficiencies or metabolic dysfunction [70].

Mechanistic understanding: While the association between homocysteine and subfertility is recognized, detailed mechanistic studies are needed to elucidate how homocysteine impacts reproductive health at the cellular and molecular levels. Investigating specific pathways, such as endothelial dysfunction, oxidative stress, hormonal disruption, inflammation, and DNA methylation, can provide critical insights. Cellular and animal models may be valuable in dissecting the molecular mechanisms linking homocysteine to subfertility, ultimately uncovering potential therapeutic targets [45].

Dose-response relationships: Exploring dose-response relationships between serum homocysteine levels and subfertility risk can provide a more nuanced understanding of the association. This investigation should involve stratifying subfertility patients into different homocysteine level categories and assessing the incidence and severity of subfertility. Dose-response analyses can help identify critical thresholds beyond which elevated homocysteine levels may significantly impact reproductive health, guiding clinical decision-making and intervention strategies [71].

Longitudinal studies: Conducting long-term prospective studies that follow subfertility patients from diagnosis through treatment and pregnancy outcomes is essential to gain insights into the impact of homocysteine over time. Such studies can elucidate whether fluctuations in serum homocysteine levels during the subfertility journey correlate with changes in fertility outcomes. Longitudinal data can also shed light on the persistence of elevated homocysteine levels and their potential influence on reproductive success, including live birth rates, pregnancy complications, and neonatal outcomes [72].

Potential Studies Needed to Validate Findings

Randomized controlled trials (RCTs): Well-designed RCTs are essential to investigate the impact of nutritional interventions targeting homocysteine levels on subfertility outcomes. These trials should assess key endpoints such as time to conception, live birth rates, and pregnancy complications. By comparing different interventions, dosages, and durations, RCTs can provide valuable insights into the efficacy and safety of interventions to normalize homocysteine levels in subfertility patients. Randomization helps control for confounding variables and ensures robust evidence [73].

Large-scale cohort studies: Large-scale cohort studies with diverse populations are crucial for providing robust and generalizable evidence regarding the relationship between serum homocysteine levels and subfertility. These studies should encompass various demographic groups, accounting for potential confounders and modifiers such as age, sex, lifestyle factors, and underlying health conditions. Long-term follow-up is essential to assess the impact of elevated homocysteine on subfertility and reproductive outcomes over time. Large cohorts can also facilitate subgroup analyses, allowing for a deeper exploration of specific risk factors and potential interactions [74].

Meta-analyses: Meta-analyses of existing studies can play a pivotal role in consolidating evidence and providing more precise estimates of the association between homocysteine and subfertility. By synthesizing data from multiple studies, meta-analyses can identify patterns and trends that may not be apparent in individual studies alone. This approach also helps quantify the strength and consistency of the association while addressing potential publication bias. Meta-analyses can guide researchers and clinicians in assessing the overall impact of homocysteine levels on subfertility and inform clinical decision-making [75].

Biological and experimental studies: Complementary to clinical research, in vitro and animal studies are essential for gaining mechanistic insights into how homocysteine affects reproductive health. These studies can help uncover the cellular and molecular pathways influenced by elevated homocysteine levels. In vitro experiments allow researchers to manipulate conditions and directly assess the impact of homocysteine on reproductive cells and tissues. Animal studies allow for exploring causal relationships, potential therapeutic targets, and the effects of homocysteine on reproductive outcomes under controlled conditions. The findings from such studies can guide future clinical research and intervention strategies [7].

Emerging Technologies and Techniques for Research

Metabolomics: Metabolomics, a high-throughput approach to analyzing small molecules in biological samples, holds immense potential for unraveling the metabolic pathways affected by elevated homocysteine levels and their association with subfertility. Researchers can identify specific metabolic perturbations linked to homocysteine dysregulation by profiling metabolites in subfertility patients. Furthermore, metabolomics can reveal potential biomarkers associated with subfertility, aiding in early diagnosis and treatment monitoring. This approach offers a comprehensive view of the metabolic landscape, providing insights into the systemic impact of homocysteine on reproductive health [76].

Epigenetics: Epigenetics, the study of heritable changes in gene expression that do not involve alterations in the DNA sequence, offers a promising avenue for investigating subfertility. Homocysteine has been implicated in epigenetic modifications, particularly DNA methylation patterns. Research on epigenetic changes in subfertility patients, influenced by homocysteine levels, can shed light on underlying molecular mechanisms. By deciphering how homocysteine impacts epigenetic regulation, researchers may uncover key genes and pathways involved in subfertility, providing novel targets for intervention and potential biomarkers for risk assessment [77].

Genomic studies: Genomic studies, including genome-wide association studies (GWAS) and targeted sequencing, can help identify genetic variations that influence homocysteine metabolism and its impact on reproductive health. Understanding the genetic underpinnings of individual responses to homocysteine dysregulation is critical for tailoring treatment approaches. Genomic studies may reveal genetic markers associated with elevated homocysteine levels and susceptibility to subfertility, paving the way for more precise risk assessment and personalized interventions. Additionally, such studies can uncover genetic factors contributing to nutrient metabolism, shedding light on how genetic variations interact with nutritional status to influence homocysteine levels [78].

Precision medicine approaches: Precision medicine, characterized by individualized treatment strategies based on a patient's genetic and metabolic profile, holds great promise in subfertility management. By integrating data from metabolomics, epigenetics, genomic studies, and clinical parameters, healthcare providers can develop tailored interventions for subfertility patients. Precision medicine allows for identifying optimal treatment modalities, dosages, and duration, maximizing the chances of successful conception while minimizing potential side effects. As our understanding of the interplay between homocysteine and subfertility deepens, precision medicine approaches can revolutionize how subfertility is diagnosed and treated, offering a new era of personalized care [79].

Importance of Personalized Medicine in Subfertility Treatment

Individualized treatment plans: Subfertility is a heterogeneous condition with diverse underlying causes. By tailoring treatment plans based on a patient's unique characteristics, including serum homocysteine levels, nutritional status, and genetic profile, healthcare providers can optimize therapeutic outcomes. For example, a patient with elevated homocysteine levels due to vitamin B12 deficiency may require a different treatment approach than a patient with a different etiology. Personalization ensures that interventions address the specific factors contributing to subfertility in each individual, potentially leading to more effective and efficient treatments [80].

Risk prediction: Developing risk prediction models that incorporate serum homocysteine levels and other relevant clinical and genetic factors can aid in identifying individuals at a higher risk for subfertility. This proactive approach allows for early intervention and tailored counseling, helping individuals make informed decisions about family planning and fertility preservation. Furthermore, it enables healthcare providers to allocate resources more efficiently, ensuring that those at greatest risk receive specialized care [81].

Nutritional guidance: Nutritional deficiencies, particularly folate and vitamin B12, can significantly impact serum homocysteine levels and subfertility. Personalized medicine in subfertility treatment involves providing targeted nutritional guidance to correct these deficiencies. For instance, individuals with specific genetic variations that impair folate metabolism may require higher folate intake or alternative forms of folate supplementation. This individualized approach ensures that nutrient deficiencies are addressed effectively, enhancing the chances of successful conception and a healthy pregnancy [82].

Monitoring and adjustments: The journey to conception can be dynamic, with various factors influencing a patient's response to treatment. Personalized medicine in subfertility management includes continuous monitoring of serum homocysteine levels and other relevant parameters. Regular assessments allow healthcare providers to track progress and adjust treatment strategies. For example, if serum homocysteine levels remain elevated despite supplementation, treatment plans can be adapted to explore alternative interventions or dosages. This iterative approach enhances the precision of care, ensuring that subfertility treatments remain tailored to the individual's evolving needs [9].

## Conclusions

This review delves into the intricate link between serum homocysteine levels and subfertility, shedding light on an understudied aspect of reproductive health. We underscore the association between elevated homocysteine and subfertility, emphasizing the role of nutrient deficiencies in this connection. Recognizing the impact of specific nutrients on homocysteine levels is crucial for improving diagnostic accuracy and treatment outcomes in subfertility. Beyond subfertility, our research emphasizes the broader implications for reproductive health, stressing the importance of considering metabolic and nutritional factors alongside hormonal aspects. A clear call to action emerges: sustained research efforts, rigorous clinical trials, personalized medicine approaches, and multidisciplinary collaboration are imperative. These endeavors will advance our understanding of subfertility, paving the way for more effective care tailored to individuals and couples facing these challenges. Committing to continued exploration, innovation, and collaboration is essential as we strive to improve reproductive health outcomes. This ensures that our evolving understanding of subfertility translates into tangible clinical practice and advancements in patient care. The goal is to provide hope for those navigating the intricate journey to parenthood.
